# Global Patterns of Net Ecosystem Exchange in peatlands: A Systematic Review and Meta-analysis of Drivers Across Land Use and Environmental Gradients

**DOI:** 10.1007/s00267-026-02621-y

**Published:** 2026-09-12

**Authors:** Charuni Jayasekara, Catherine Leigh, Jeff Shimeta, Ewen Silvester, Samantha Grover

**Affiliations:** 1 Department of Applied Chemistry and Environmental Science, School of Science, RMIT University, Melbourne, VIC Australia; 2 School of Agriculture, Food and Ecosystem Sciences (SAFES), Faculty of Science, The University of Melbourne, Parkville, VIC Australia; 3 School of Biology and Environmental Science (BES), Queensland University of Technology, Brisbane, QLD Australia; 4 Department of the Environment, Tourism, Queensland Herbarium and Biodiversity Science, Science and Innovation, Brisbane, QLD Australia; 5 Department of Biology, School of Science, RMIT University, Bundoora, VIC Australia; 6 Department of Ecological, Plant and Animal Sciences, Research Centre for Applied Alpine Ecology, La Trobe University, Wodonga, VIC Australia

**Keywords:** CO_2_ flux, Degradation, Peatland condition, Water table depth, Restoration, Net Ecosystem Exchange (NEE)

## Abstract

Peatlands play an essential role in the global carbon cycle, storing approximately one-third of the world’s soil carbon despite covering less than 3% of the land surface. Peatland degradation from anthropogenic activities and climate change can convert peatlands from net carbon sinks to sources by altering carbon cycling. Net Ecosystem Exchange (NEE), the balance between CO_2_ uptake and emission, is a critical indicator for assessing peatland condition and restoration efforts. We conducted a systematic quantitative literature review to investigate global patterns of NEE in peatlands and identify key environmental and anthropogenic drivers of CO_2_ flux variability. Annual NEE values from 120 globally distributed sites reported in peer-reviewed literature were analyzed in relation to climatic zone, land use, vegetation type, peatland condition, and water table depth. Our synthesis revealed significant geographic gaps, with peatland NEE studies substantially underrepresented in the Tropics, Africa, and Oceania. Agricultural peatlands emitted significantly more CO_2_ than sites under natural land uses or peat extraction, while degraded peatlands were significantly greater net CO_2_ sources than intact and restored systems. Restored peatlands remained net CO_2_ sources on average, emphasizing the importance of long-term monitoring and adaptive management following restoration interventions. Water table depth significantly affected NEE variability, with CO_2_ emissions increasing approximately 7.2 gCO_2_-C m^−2^yr^−1^ for every centimeter of water table drawdown. A substantial variability in measurement methods, data processing software, and protocols highlighted the critical need for methodological standardization. Our findings provide evidence-based targets for peatland conservation and restoration monitoring as nature-based climate solutions.

## Introduction

Peatlands represent one of Earth’s most significant terrestrial carbon reservoirs, occupying 2.84% of the planet’s land surface (Xu et al. 2018) yet storing an estimated ~ 600–942 Gt of carbon globally, the largest amount of carbon per unit area of all ecosystems (Widyastuti et al. 2025; Yu et al. 2010). These terrestrial wetland ecosystems, particularly in the Northern Hemisphere, began forming 8,000-12,000 years ago following deglaciation and subsequent sea-level rise at the end of the last ice age, establishing under conditions of positive climatic moisture balance and waterlogged topography (MacDonald et al. 2006). However, peatlands have repeatedly established and persisted over at least the last 130,000 years (Treat et al. 2019), while some tropical peatlands are considerably older than their high-latitude counterparts (Ruwaimana et al. 2020). The unique combination of consistently high water tables for the majority of the year, low substrate pH, and limited nutrient availability creates anaerobic conditions that suppress microbial decomposition, enabling the net accumulation of organic matter (Couwenberg et al. 2009; French et al. 2016; Page et al. 2006) at long-term rates of approximately 20–30 gC m^−2^ yr^−1^ (Turunen 2003). As a result, natural or intact peatlands absorb more carbon through primary productivity than they emit through decomposition, making them net carbon sinks (Gorham 1991; Joosten 2010; Waddington et al. 2014).

The critical carbon storage function of peatlands is increasingly threatened by anthropogenic disturbances (UNEP 2022). Approximately 65 million ha (16%) of the world’s peatlands have been drained to enable alternative land uses (e.g., agriculture, forestry) (Joosten 2010; UNEP 2022), thereby fundamentally altering their hydrological regimes and carbon dynamics (Joosten 2015). Artificial drainage lowers water table levels, leading to accelerated peat oxidation processes (Jayasekara et al. 2024), converting these ecosystems from carbon sinks to sources through increased aerobic decomposition of stored organic matter (Couwenberg et al. 2009). Degraded peatlands were estimated to account for 3% of the world’s anthropogenic GHG emissions in 2020 (UNEP 2022), with ~2 Gt CO_2_-eq GHGs emitted annually, and this amount was expected to increase up to 37–174 Gt by 2100 (Maljanen et al. 2010; Schuur et al. 2015; Urák et al. 2017). This escalating contribution to global greenhouse gas budgets has prompted extensive management initiatives by international organisations, including the European Union (EU), Food and Agricultural Organisation (FAO), International Union for the Conservation of Nature (IUCN), and United Nations Framework Convention on Climate Change (UNFCCC) (Joosten 2015; Joosten et al. 2012; UNEP 2022).

Central to understanding and managing peatland carbon dynamics is Net Ecosystem Exchange (NEE) (Baldocchi 2003), which quantifies the net CO_2_ flux between ecosystems and the atmosphere. NEE represents the difference between Gross Primary Production (GPP, the total CO_2_ fixation by autotrophs) and Ecosystem Respiration (ER, the total CO_2_ release through autotrophic and heterotrophic respiration); thus, NEE = -GPP + ER (Chapin et al. 2006). Studies employ multiple methods to measure NEE, primarily eddy-covariance and chamber methods (Poyda et al. 2017; Schrier-Uijl et al. 2010; Wang et al. 2010). Eddy covariance method offers continuous ecosystem-scale measurements capturing temporal dynamics and inter-annual variability, using software tailored to process high-frequency data, while chamber-based approaches provide crucial insights into spatial heterogeneity and process-level mechanisms, using standard statistical software (Baldocchi 2003; Fang and Moncrieff 1996). Despite decades of peatland carbon flux research employing both methodologies, NEE data remain fragmented across studies varying in spatial scale, temporal duration, measurement protocols, and reporting standards.

Peatlands exhibit distinct characteristics under different regional, geographic, and climatic conditions, resulting in substantial variation in their physical, chemical, and biological properties, thus affecting CO_2_ cycling (Blodau 2002; Limpens et al. 2008; Waddington et al. 2014). Peatland classification systems vary globally, with temperate and boreal regions distinguishing between bogs (ombrotrophic, rain-fed systems) and fens (minerotrophic, groundwater-fed systems), while tropical and subtropical regions often employ broader peatland categorizations (Minasny et al. 2019; Xu et al. 2018). Vegetation structure ranges dramatically from *Sphagnum* moss-dominated systems in boreal regions to forested tropical peatlands supporting tall tree canopies, with corresponding differences in primary productivity, litter quality, and decomposition rates (Crow and Wieder 2005; Jayasekara et al. 2024; Ward et al. 2009). Additional physical variability in peat depth, ranging from shallow (<1 m) deposits to deep (>10 m) accumulations, surface area, hydrological connectivity, and water chemistry (pH, nutrient availability, dissolved organic matter composition) (Crow and Wieder 2005; Strack and Waddington 2007) further contributes to heterogeneity in carbon-cycling processes (Mander et al. 2025). These structural and functional differences translate into substantial variation in carbon accumulation rates across climatic zones (Jayasekara et al. 2025a; Mander et al. 2025). For example, tropical peatlands demonstrate higher carbon accumulation rates (59–145 gC m^−2^ yr^−1^) (Ribeiro et al. 2021; Sorensen 1993) than their temperate, boreal, and sub-arctic counterparts (30–35 gC m^−2^ yr^−1^) (Khodaei et al. 2023; Turunen 2003). Despite the critical importance of peatlands in global carbon cycling and the recognized heterogeneity, significant knowledge gaps remain regarding how NEE variability relates to peatland type, geographic location, climatic conditions, vegetation characteristics, and management states across global gradients.

The lack of systematic synthesis limits our ability to establish robust baseline CO_2_ flux estimates for different peatland types and management conditions, identify generalizable patterns across spatial and temporal scales, determine critical thresholds and prioritize conservation and restoration investments (Joosten 2015; UNEP 2022). Furthermore, with increasing international commitments to peatland restoration under climate mitigation strategies, evidence-based management targets, particularly regarding critical water table depths, recovery timeframes, and intervention effectiveness, are urgently needed to guide policy and practice (FAO 2020; Strack et al. 2022; UNEP 2022). This research addresses the need for a comprehensive synthesis of global peatland CO_2_ flux data by synthesizing global patterns of net ecosystem exchange (NEE) in peatlands across different ecosystem states, management practices, and environmental conditions and identifying the key environmental and anthropogenic drivers of CO_2_ flux variability. The specific objectives of our study are to (i) identify the global distribution and scope of peatland NEE research, (ii) quantify baseline peatland NEE patterns across climatic zones, peatland types and conditions, (iii) evaluate the influence of disturbance and management on peatland CO_2_ exchange, (iv) determine the role of water table depth in controlling NEE variability, and (v) synthesize evidence-based recommendations for peatland management and restoration.

## Methods

### Literature Search

**Fig. 1:**
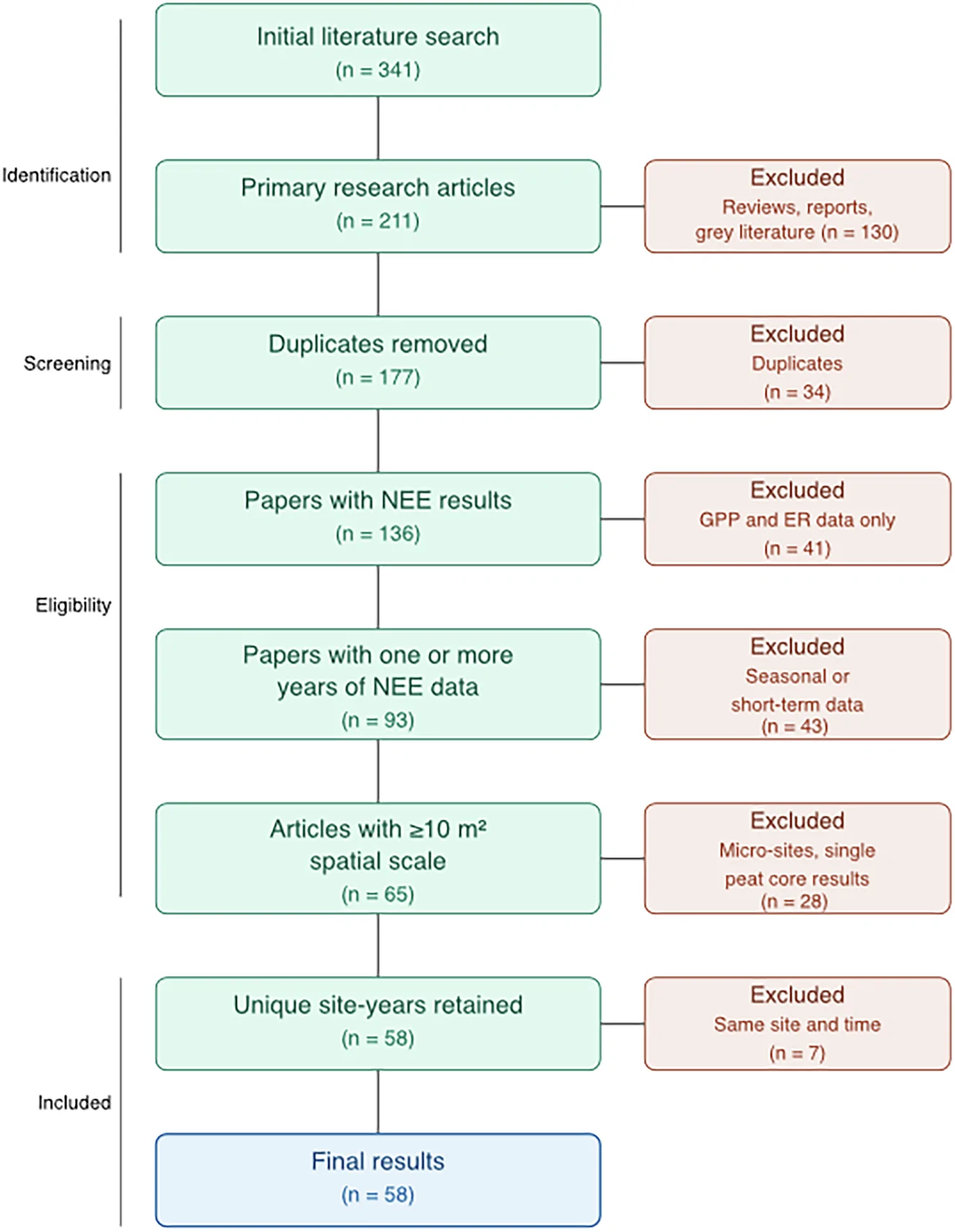
PRISMA flow diagram illustrating the inclusion and exclusion criteria applied during the quantitative literature review, resulting in 58 articles for analysis

This systematic quantitative literature review was conducted between March 2021 and August 2021 following the method outlined by Pickering and Byrne (2014). We identified relevant literature using keyword searches in electronic databases that index scientific, peer-reviewed journals (Web of Science, Science Direct, Scopus, SciFinder, Pro-Quest and Google Scholar) across all years of available records. The principal keywords “peat” and “net ecosystem exchange” were used in combination with “mire, bog, fen, wetland, swamp, NEE, net CO_2_ exchange, CO_2_ emission*, carbon cycling, carbon balance, degrad*, natural, restore, agricultur*, tropical, northern hemisphere, and southern hemisphere”. The initial literature search yielded 341 articles (Fig. 1).

We removed review articles, gray literature, duplicates, as well as studies conducted at small spatial scales (in plots of less than 10 m^2^) to ensure sufficient spatial representation of the peatland area. We excluded laboratory studies as they do not capture field conditions, and they ignore the effect of vegetation. We excluded field manipulation studies in which NEE was measured after sites had been experimentally manipulated. We also excluded short-term studies with no annual NEE values, given that a net balance should be representative of at least a one-year temporal scale. The selected articles included studies that measured NEE all 365 days of the year, plus studies that measured NEE multiple times a year and extrapolated for the whole year. We also included cumulative NEE for all four seasons of one year but excluded studies that only included Net Ecosystem Carbon Balance (NECB), GPP or ER and those reporting only daytime NEE.

For studies reporting NEE values across multiple years, we used the site mean NEE averaged across all available years, as per established protocols (Gong et al. 2020; McLaughlin and Webster 2018; Webster et al. 2018). This approach characterizes the general carbon balance of each site rather than capturing interannual variability. Annual carbon budgets are also the standard metric for climate policy frameworks, including national greenhouse gas inventories, and restoration and conservation management decisions.

### Database Creation

In this study, any peatland site for which a NEE value was described and reported was considered a separate site. As such, some articles included more than one site. For each site, we recorded information on the journal article in which it was described, including author name(s), title, year published, the journal and its major discipline. The recorded regional settings for each site included hemisphere (northern or southern), continent (Asia, North America, South America, Europe, Africa, and Oceania), and country. For the purposes of this review, mainland USA and Alaska were recorded separately due to their differences in climate and geographical location.

The methods used to measure NEE were categorized as Eddy Covariance (EC), Chamber (chambers placed over the soil surface connected to a gas analyser to quantify CO_2_ exchange) and ‘other’. We recorded the software used to process raw CO_2_ data (given that different software uses different quality control and gap filling techniques), number of measurement years, annual NEE values and each site’s carbon sink and source status as reported in the relevant article. The latter was recorded as per convention in the environmental science and ecology disciplines, as defined by Chapin et al. (2006), where a net carbon sink is represented by a negative NEE value and a net carbon source by a positive NEE value. Sites were categorized into six climate sub-categories: polar, sub-polar, temperate, alpine, dry, and tropical zones, together with the mean annual temperature and precipitation at the site, as reported in the articles. The following physical characteristics were recorded: peat depth, peat area and elevation of the site. Where elevation was not reported we used coordinates to estimate site elevation in the “Google Earth” (http://earth.google.com) application. We also extracted the average water table depth of sites when available, but many studies did not account for surface oscillations in their measurements; therefore, water table depth is not fully standardized across sites.

Sites were classified according to peatland type (bog, fen, peatland, mire, wetland, or peat swamp forest) and vegetation categories based on descriptions in the articles. The vegetation types varied substantially among sites, and we identified six major types: tree dominant, shrubs/grasses, moss dominant, pasture, crops, and plantations, as well as sites with bare peat. Next, these vegetation types were categorized into land uses as native (tree, shrubs/grasses, and moss dominant vegetation), agricultural (pasture, crops, and plantations) and bare peat. The condition of the peatlands was classified based on descriptions in the articles as: “intact” (natural, near natural, or peatlands that had recovered nearly to natural condition as described as such in the articles), “restoration” (peatlands that were undergoing any kind of rehabilitation or revegetation activity), or “degraded” (peatlands that had experienced any form of anthropogenic disturbance such as agriculture, artificial drainage, mining, or grazing).

### Data Analysis

Data analysis was conducted using base packages in the open-source R Statistical Software version 4.4.1 (R Core Team 2020). Summary statistics (mean, median, standard deviation (SD) and standard error (SE)) were calculated for NEE, peat depth, area, site elevation, mean annual temperature, precipitation and water table depth. NEE data were grouped by land-use category, vegetation type, climatic zone, and peatland condition to assess variability in CO_2_ flux across ecosystem states and environmental conditions. We used the Shapiro-Wilk test to assess the normality of the NEE dataset, which indicated that the NEE data were not normally distributed. Consequently, we employed non-parametric statistical tests for all categorical analyses. The Scheirer-Ray-Hare test was used to investigate the interactive effects of land use and vegetation categories on NEE, while the Kruskal-Wallis test was applied to examine the separate effects of land use and vegetation on NEE. Post-hoc pairwise Dunn tests with Bonferroni correction were used to identify significant differences between individual categories. Parametric linear regression analysis and segmented (breakpoint) regression (Online Resources 1) were used to examine the relationship between water table depth and NEE, and model assumptions were verified using residual diagnostics.

## Results

### Current State of The Global Peatland NEE Literature

**Fig. 2:**
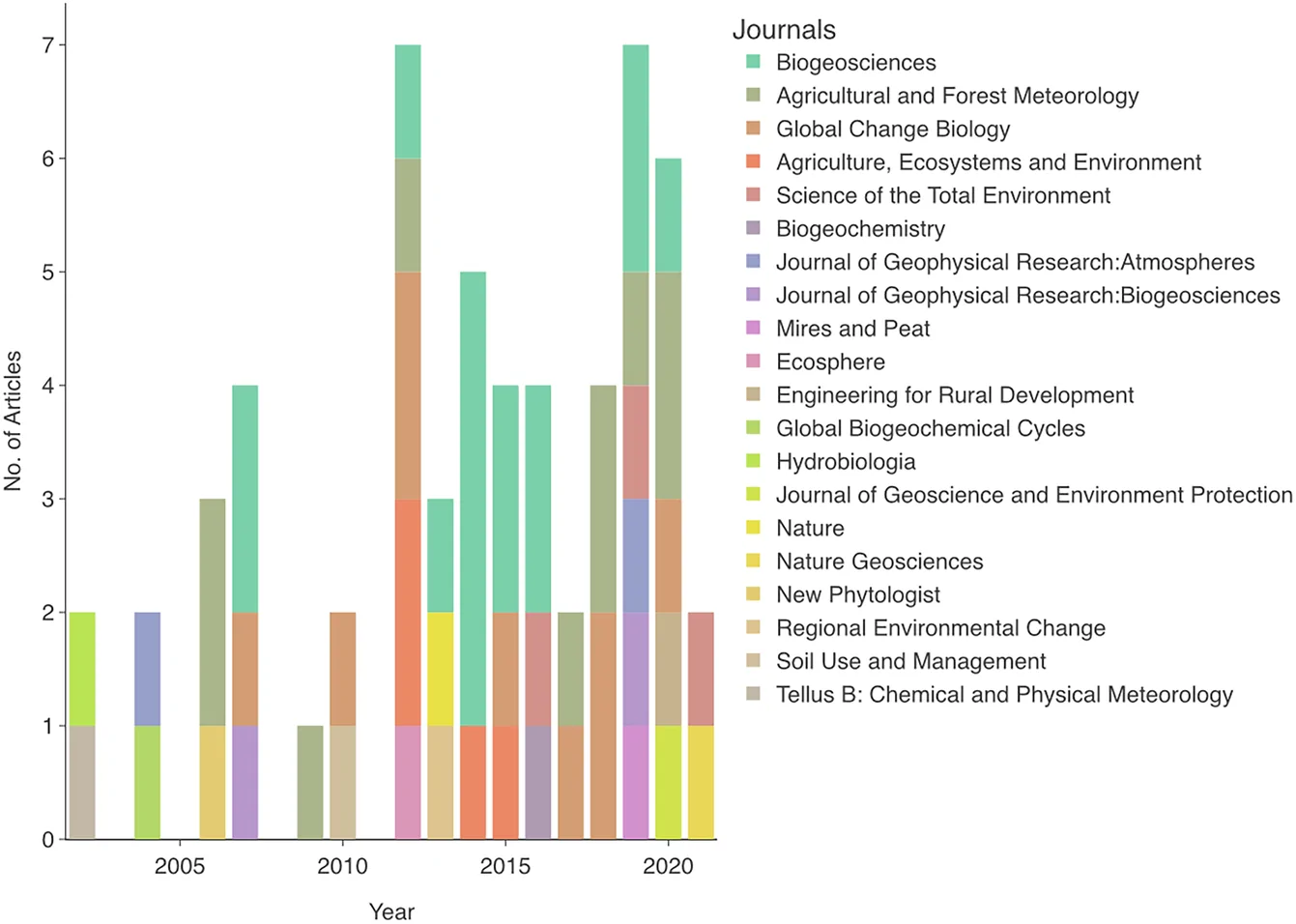
Number of peatland NEE articles published in each year, grouped by journal

Fifty-eight primary, peer-reviewed research articles that reported long-term NEE values published in 20 different English language journals fulfilled the selection criteria for this systematic quantitative review. All the articles were published after 2002. The highest annual number of articles was published in 2012 and 2019 (seven each), followed by 2020 (six papers; Fig. 2). About 75% of the articles were published after 2010, with no articles published in 2003, 2005, or 2008.

The journal *Biogeosciences* published the greatest number of articles (14), followed by *Agricultural and Forest Meteorology* (10) and *Global Change Biology* (9). The remaining 18 journals published 45% of the articles, encompassing a broad range of disciplines, including ecology, agriculture, environmental science, geology, and biochemistry.

### Distribution of Peatland Studies

**Fig. 3:**
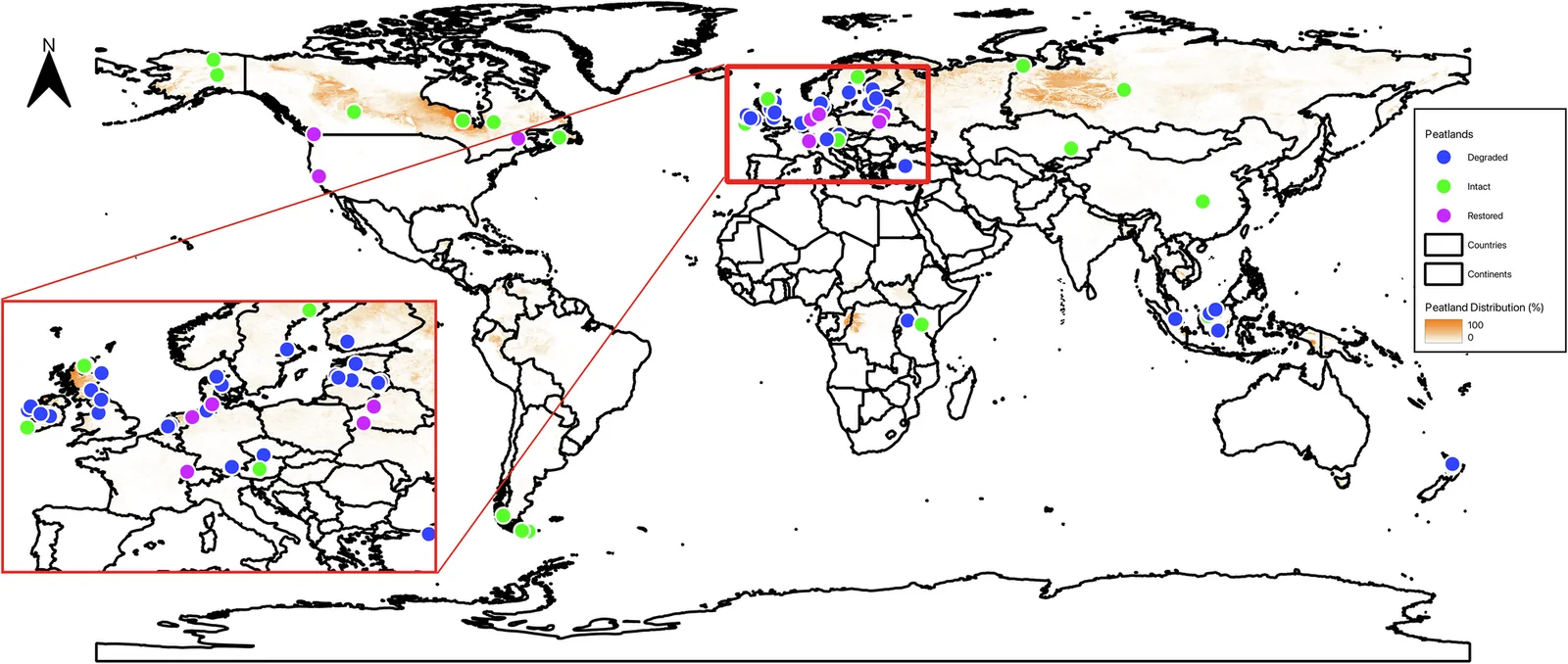
Geographic distribution of 120 peatland NEE sites categorized by peatland condition and overplotted on the global peatland distribution map by Melton et al. (2022). The inset shows the details of European sites where study density is highest

Of the 58 articles, we identified 120 peatland sites; many selected articles examined more than one peatland or multiple sites within the same large peatland. Peatland NEE was measured across both hemispheres, all continents (excluding Antarctica) and twenty-eight countries (Fig. 3). Only 14% (16) of the selected sites were in the Southern Hemisphere, whereas the remainder (104) were in the Northern Hemisphere. The greatest number of studies were conducted in Europe (60%), where Germany (13), Denmark (11), Ireland (11), and Latvia (8) had most study sites. North America had the second-highest number of sites (24) as a continent, with 14 in Canada and ten in the USA (including Alaska), while Asia had 13 sites, with six of these in Indonesia. Oceania had six sites, while both Africa and South America had three study sites.

### Characteristics of The Studied Peatlands

We identified 42 sites that were described as bogs, 14 as fens, 12 as mires, and 10 as peat swamp forests. However, 36 sites were solely described as “peatlands”, while 6 were referred to only as “wetlands.” The sites were located across a range of different elevations from 14 m below to 3460 m above sea level. The depth of the peatlands ranged from 0.02 m (a highly impacted peat mining site) to 13 m, while the mean peat depth was 3.1 ± 2.8 m. The peatland site areas ranged from 0.01 to 366 km^2^, with a mean of 29 km^2^. With regard to meteorological parameters, the mean annual temperature of the sites ranged from −7.5 °C to 27 °C, and the mean annual precipitation ranged from 41 to 3000 mm.

### Data Collection and Processing Methods

The period over which NEE measurements were taken ranged from one year (43 sites) to over five years (5 sites), with the longest measurement time being 17 years. About half of the selected sites (63) measured NEE over a period of two to three years, while 10 sites had measurements for a period of four to five years. Chamber (64 sites) and EC methods (54 sites) were used to measure NEE nearly equally often, with one study using both methods, and only two sites using other methods (peat cores).

A wide range of software was used to analyze the peatland NEE data, with processing of the EC method data using 15 different software programs and processing of the chamber method data using seven. About 40% of the studies that used EC data were processed using EddyPro software, followed by MATLAB and EddyProc at 15% and 10%, respectively. SPSS software was used to process about 25% of the chamber data, while MATLAB, Minitab, and R had relatively similar usage rates of 20%, 19%, and 17%, respectively.

### NEE Variability Across Climatic Zones

**Fig. 4:**
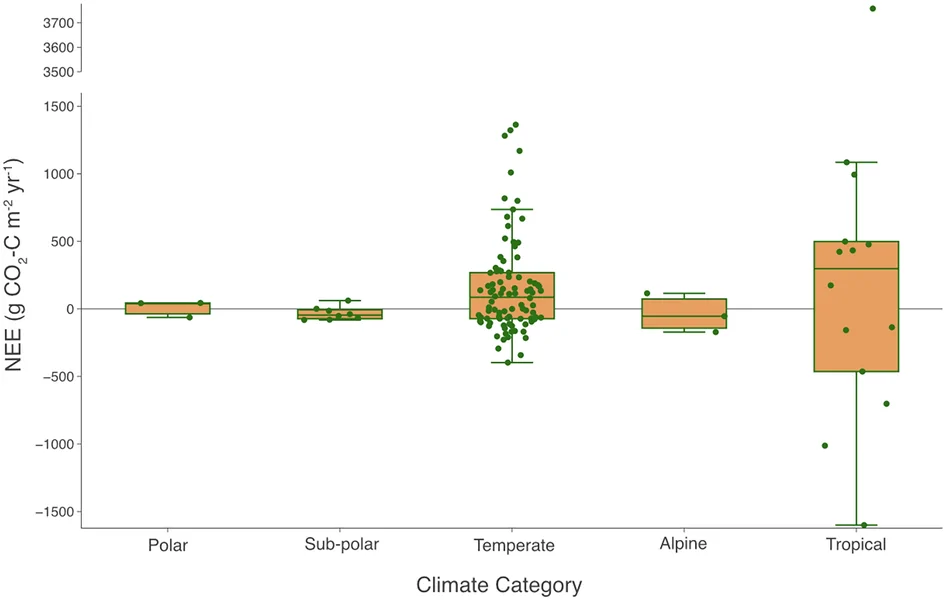
Variation in annual net ecosystem exchange (NEE) among peatlands across different climatic zones

About 77% (92) of the studied sites were from the temperate climate zone, while only 12% (14) were in the tropics. There were eight sub-polar sites, and three sites each in alpine and polar climate zones. NEE differed considerably among climatic zones (Fig. 4), with peatlands in temperate, tropical, and polar zones across all land uses being (mean) net CO_2_ sources, while alpine and sub-polar zones were net CO_2_ sinks. Alpine and sub-polar zone peatlands were the strongest mean net CO_2_ sinks (−37 ± 144 and −34 ± 17 gCO_2_-C m^−2^ yr^−1^, respectively), while tropical peatlands were the greatest mean net CO_2_ sources ( +270 ± 1255 gCO_2_-C m^−2^ yr^−1^) and had the greatest variation in NEE.

### Land Use and Vegetation Effects on NEE

**Fig. 5:**
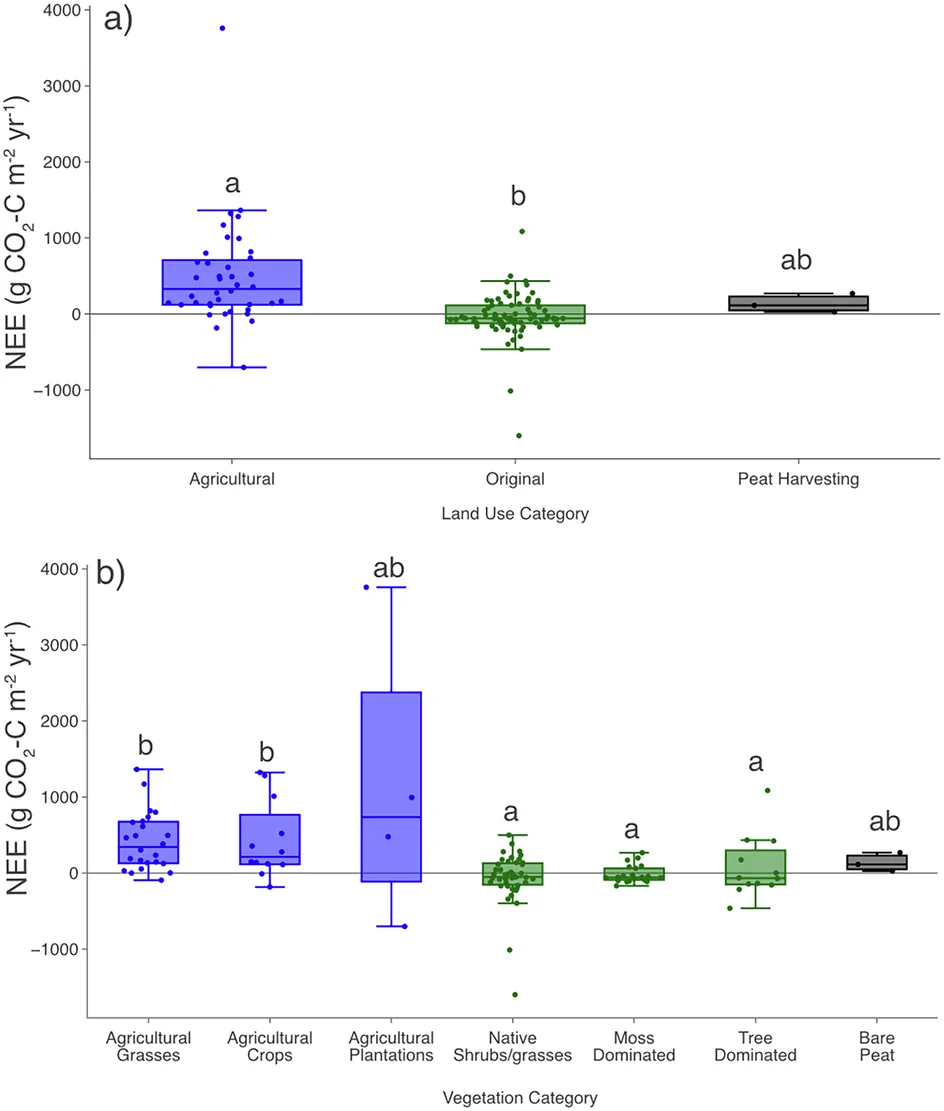
Annual net ecosystem exchange (NEE) of peatlands across (**a**) land use categories and **b** vegetation types. In **b**, colors represent land use: blue = agricultural, green = original, gray = peat harvesting. Different letters denote statistically significant differences between groups

Categorizing sites according to their land uses resulted in three main categories (Fig. 5a). About 64% (77) of sites had not undergone land use change (we refer to these sites as having ‘original’ land use), while 40 sites had agricultural use, and three sites were used for peat harvesting. We note that ‘original’ here denotes the absence of a documented land-use change rather than an undisturbed history, as legacies of past disturbance could not be fully reconstructed from the source articles. Both agricultural and peat-harvesting land use categories were net CO_2_ sources with mean CO_2_ emission values of +490 ± 693 and +137 ± 123 gCO_2_-C m^−2^ yr^−1^, respectively. In contrast, sites with original land use were net CO_2_ sinks with a mean net CO_2_ absorption value of −30 ± 307 gCO_2_-C m^−2^ yr^−1^. Land use had a significant effect on NEE (*p* < 0.001), with CO_2_ emissions in agricultural land uses being significantly higher than in original land uses (*p* < 0.001). There were no significant differences in NEE between agricultural and peat-harvesting land uses (*p* = 0.5), nor between original and peat-harvesting land uses (*p* = 0.3).

The three land use categories were further classified into 7 different vegetation categories (Fig. 5b). Forty-three of the original land use sites had native shrubs/grasses, 22 were moss-dominant, and 12 had tree-dominant vegetation type. From the agricultural land use category, 24 sites had pasture as the dominant vegetation, 12 had crops, and four had plantations. The three peat-harvesting sites had no vegetation and, therefore, were categorized as bare-peat sites. All the agricultural vegetation sites were net CO_2_ sources, with plantations having the highest mean net CO_2_ emission values (+1132 ± 1890 gCO_2_-C m^−2^ yr^−1^). Pasture and crops emitted mean net CO_2_ amounts of +416 ± 382 and +424 ± 507 gCO_2_-C m^−2^ yr^−1^, respectively. From the peatland sites with original land uses, tree-dominant vegetation sites were net CO_2_ sources with a mean NEE value of +72 ± 410 gCO_2_-C m^−2^ yr^−1^. However, both the native shrub/grasses and moss-dominated vegetation categories were net CO_2_ sinks with mean net CO_2_ absorption values of −65 ± 341 and −18 ± 114 gCO_2_-C m^−2^ yr^−1^, respectively. The NEE of vegetation categories differed significantly from each other (*p* < 0.001). Agricultural pasture vegetation had significantly higher CO_2_ emissions than moss-dominated, tree-dominated, and natural shrub/grasses vegetation (*p* < 0.001 on all occasions), while agricultural crops had significantly higher CO_2_ emissions than moss-dominated and natural shrub/grasses vegetation (*p* < 0.001 on all occasions). There were no significant interactions between land use and vegetation categories within each land use group (*p* = 0.98).

### Peatland Condition and Restoration Outcome

**Fig. 6:**
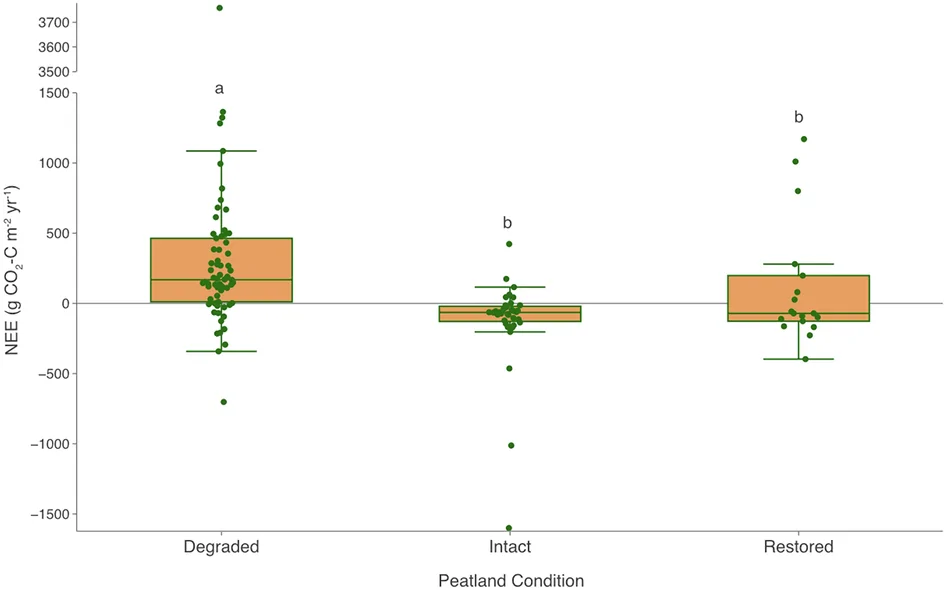
Annual net ecosystem exchange (NEE) of peatlands across peatland condition categories. Different letters denote statistically significant differences between groups

The peatland condition analysis indicated that about 55% of our sites (66) were impacted by various anthropogenic activities (in ‘degraded’ condition); about 30% were categorized as ‘intact’ (36), and 15% (18) had some past or current restoration activities (‘restored’) (Online resources 1, Table S1). The categorization of peatlands according to their condition showed that degraded peatlands were mean net CO_2_ sources ( + 306 ± 578 gCO_2_-C m^-2^ yr^-1^), while the intact peatlands were mean net CO_2_ sinks (−124 ± 327 gCO_2_-C m^−2^ yr^−1^) (Fig. 6). The restoration peatland category was also a mean net CO_2_ source with +110 ± 438 gCO_2_-C m^−2^ yr^−1^. The average NEE values of the three peatland condition categories differed significantly (*p* < 0.001), with degraded peatlands having significantly higher CO_2_ emissions than intact (*p* < 0.001) and restored (*p* < 0.001) peatland categories. There was no significant difference in NEE between intact and restored peatland categories (*p* = 0.2).

**Fig. 7:**
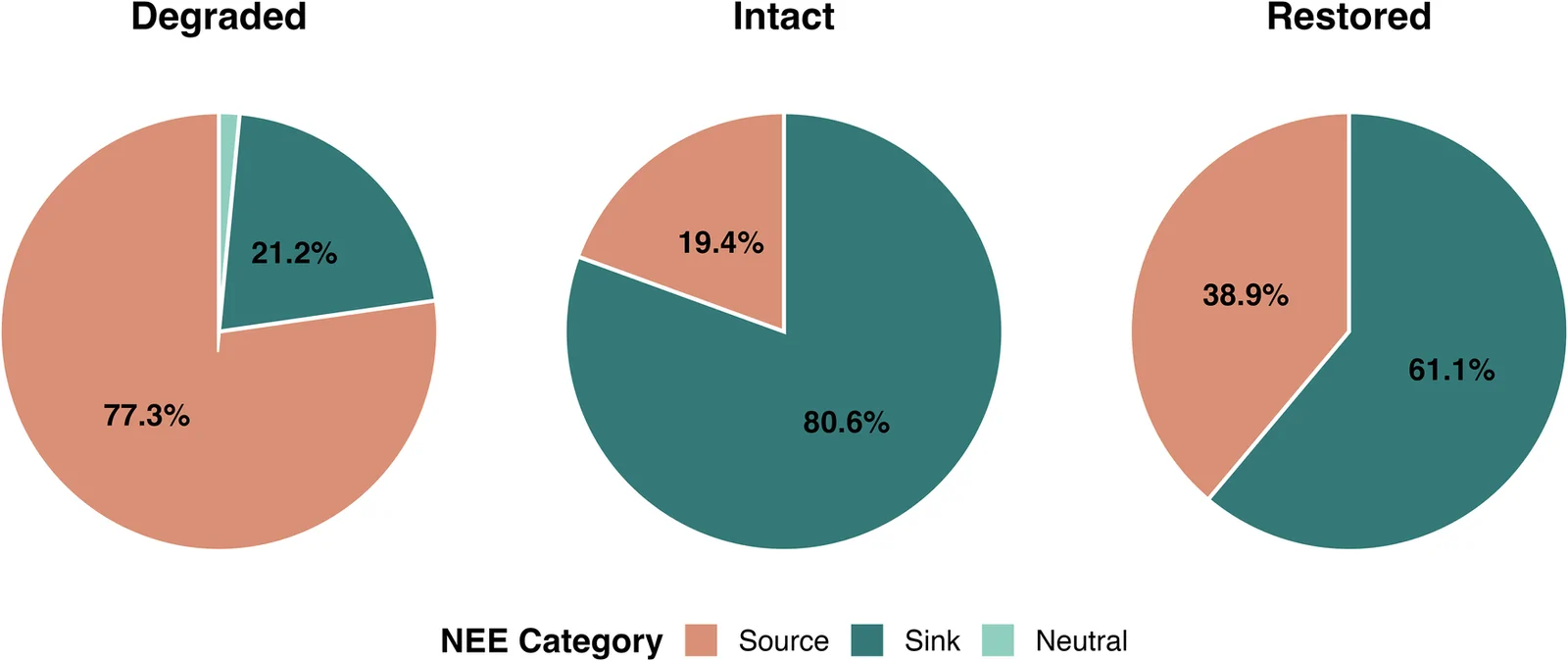
Proportion of peatland sites exhibiting net carbon sink, source, or neutral behavior across different condition categories

Overall, 65 sites were net CO_2_ sources, with the highest CO_2_ emissions of 3758 gCO_2_-C m^−2^ yr^−1^ being from a tropical oil palm plantation established on a peatland. Fifty-four sites were net CO_2_ sinks, with the highest CO_2_ absorption value in a tropical papyrus wetland (-1600 gCO_2_-C m^−2^ yr^−1^), while one site was CO_2_ neutral (0 ± 162 gCO_2_-C m^−2^ yr^−1^). However, of the sites classified as degraded, only 51 out of 66 were CO_2_ sources, while 14 remained CO_2_ sinks, and one was carbon neutral (Fig. 7). Similarly, not all sites that were categorized as intact were carbon sinks; 29 out of 36 intact sites acted as net CO_2_ sinks, and 7 were net CO_2_ sources. Out of 18 restored sites, 11 were net CO_2_ sinks, while 7 were net CO_2_ sources at the time of measurement.

### Water Table ConTrols on NEE

**Fig. 8:**
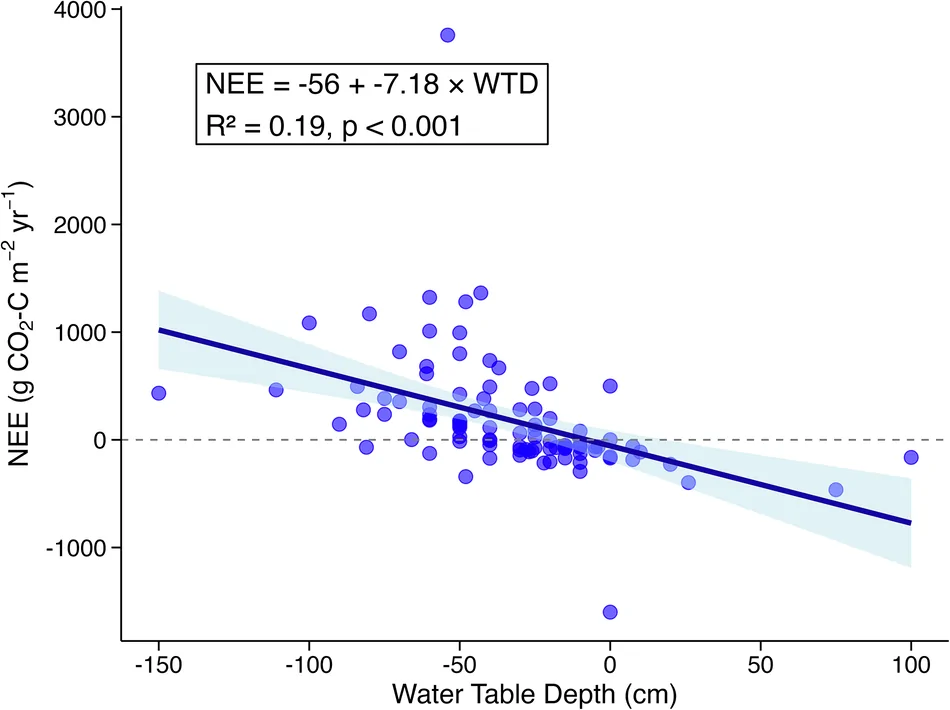
Linear regression relationship between water table depth and net ecosystem exchange (NEE) across global peatlands

Ninety-eight sites had data on water table depth, which ranged from 150 cm below the peat surface (in a heavily degraded tropical peatland) to 100 cm above the peat surface (in a restored temperate wetland). The mean water table depth of sites was 34 ± 34 cm below the peat surface, with a median value of 30 cm below the surface. There was a negative correlation between NEE and water table depth (*R*^2^ = 0.19, *p* < 0.001), indicating that deeper water tables were associated with higher CO₂ emissions (Fig. 8). There was no evidence of a water table threshold, but peatlands transitioned from a net CO_2_ sink to a source at 7.8 cm below the peat surface (Online resources 1, Fig. S1). The regression model indicated that for every 1 cm decrease in water table depth (i.e., water table drawdown), NEE increased by approximately 7.2 gCO_2_-C m^−2^ yr^−1^ (Fig. 8). This relationship explained 19% of the variance in NEE across sites, highlighting the critical role of hydrology in peatland carbon dynamics.

## Discussion

### Distribution of Peatland Studies

Our systematic review reveals a pronounced geographic bias in global peatland NEE research, with 86% of studies concentrated in the Northern Hemisphere and 60% in Europe. This distribution severely underrepresents the Southern Hemisphere, Asia, Oceania, and Africa, regions that contain substantial and threatened peatland areas. The limited number of studies from Asia and Africa may reflect technical, financial, and political constraints that impede the establishment and maintenance of long-term CO_2_ flux-monitoring infrastructure (Lawson et al. 2014; Leng et al. 2019; Sinclair et al. 2024). In cold-climate regions, logistical challenges and the need to confine measurements to the growing season (to avoid harsh winter conditions) may limit year-round monitoring capacity (Mander et al. 2025; Virkkala et al. 2017). Additionally, an unknown but potentially substantial body of research from underrepresented regions may exist in non-English language publications or gray literature and remain inaccessible to global syntheses (Leng et al. 2019; Sinclair et al. 2024). Furthermore, this geographic concentration may be compounded by the absence of a globally consistent peatland classification. The widely used bog–fen typology reflects a temperate and boreal framework, whereas tropical and subtropical peatlands are typically described using broader categories (Minasny et al. 2019; Xu et al. 2018). Inconsistent classification also directly constrained finer peatland type-based comparisons in our synthesis, as 42 of 120 sites were identified generically as “peatland” or “wetland”.

The severe underrepresentation of Asian and tropical peatland NEE research is particularly concerning, given that these regions account for the largest share of global peatland area (Xu et al. 2018), and may be most vulnerable to climate change impacts and land-use conversion, particularly in Southeast Asia (Calle et al. 2016; Miettinen et al. 2016). Our finding that tropical peatlands functioned as the strongest net CO_2_ sources ( + 270 ± 1255 gCO_2_-C m^−2^ yr^−1^) despite representing only 12% of study sites highlights the urgent need for expanded research and conservation efforts on these peatlands, especially given their rapid conversion rates for agriculture and forestry (Page et al. 2022; Ribeiro et al. 2021). Similarly, high-latitude peatlands in polar and subarctic regions require expanded monitoring, as their substantial carbon stocks are increasingly vulnerable to permafrost thaw under global warming (Väliranta et al. 2021; Voigt et al. 2019).

### NEE Patterns Across Land Use Types

Our analysis demonstrates that land-use conversion alters peatland carbon dynamics, with agricultural peatlands emitting significantly more CO_2_ than those maintained in their original land use. The large difference in CO_2_ flux between original (-30 gCO_2_-C m^−2^ yr^−1^ sink) versus agricultural land use ( + 490 gCO_2_-C m^−2^ yr^−1^ source) provides quantitative evidence for the carbon cost of peatland conversion. Agricultural utilization of peatlands typically involves intensive drainage (ditches, channels), fertilizer applications, vegetation removal, and landscape modification (Gronlund et al. 2006; Jackson et al. 2009; Schrier-Uijl et al. 2010). These changes alter peat properties and increase the dynamism of patterns in soil moisture, substrate availability, and redox activity (Carlson et al. 2012; Strack and Waddington 2007; Ward et al. 2007), leading to enhanced aerobic peat decomposition (Carlson et al. 2012; Hatano 2019; Jayasekara et al. 2024) and CO_2_ emissions (Jayasekara et al. 2025b; Oertel et al. 2016; Schrier-Uijl et al. 2014). Removal of natural vegetation and seasonal biomass harvesting eliminates year-round photosynthetic CO_2_ uptake (Hatala et al. 2012; Knox et al. 2015; Ward et al. 2007), while exposed soil surfaces during fallow periods or between crop rows further enhance decomposition rates (Marushchak et al. 2013; Väliranta et al. 2021). In contrast, peatlands under their original land use, including intact and native vegetation categories in our study, typically sustain near-surface water tables (Furukawa et al. 2005; Knox et al. 2015), low aerobic microbial biomass (Andersen et al. 2013), low CO_2_ emissions (Dise 2009), and efficient CO_2_ fixation through high above and belowground biomass growth (Fleischer et al. 2016), making them net CO_2_ sinks (Liu et al. 2019). Among sites in the agricultural land use category in our study, plantations exhibited the highest emissions ( + 1132 ± 1890 gCO_2_-C m^−2^ yr^−1^), likely reflecting prolonged growing cycles that require persistent drainage, limited ground cover, and high biomass removal at harvest (Kiew et al. 2018; McCalmont et al. 2021; Saunders et al. 2012). These findings raise the question of whether any form of “sustainable” peatland agriculture is viable from a climate perspective.

### Peatland Condition and Restoration Outcomes

Although our results largely agree with the widely accepted finding that degraded peatlands are net CO_2_ sources (Hatano 2019), we found that 19% of “intact” peatlands were net CO_2_ sources, while 21% of “degraded” peatlands were net CO_2_ sinks. This pattern suggests that traditional classification approaches based on visual assessment, hydrological modifications, or land-use history inadequately capture the functional state of ecosystems. Natural interannual variability in carbon balance, driven by climate fluctuations, means that short-term measurements (1–2 years) may not reflect long-term trajectories (Holl et al. 2019). Additionally, intact peatlands may undergo cryptic degradation through atmospheric deposition, invasive species, or climate-driven changes in the water table that disrupt carbon-sink function without visible structural changes (Bragazza et al. 2008; Limpens et al. 2008; Lu et al. 2025). Furthermore, classification schemes lacking direct carbon flux measurements or detailed hydrological monitoring may fail to detect functionally significant ecosystem state changes (Couwenberg et al. 2009). Wang et al. (2018) identified that abandoned peatland pasture had significantly higher carbon accumulation ability than a pristine bog, due to high aboveground biomass, while a drained forest bog was a stronger carbon sink than natural bog forest due to high gross primary productivity of mature trees (Hommeltenberg et al. 2014). In addition, a drainage-affected bog has been shown to have a strengthened CO_2_ sink 16 years after degradation, despite having a lowered water table, due to the new vegetation composition at the site that fixed more CO_2_ through photosynthesis than the original peatland vegetation (Ratcliffe et al. 2020). Moreover, drainage ditches can affect not only the intended peatland area but also neighboring pristine areas and may also drain out water, turning intact areas into net carbon sources (Joosten 2010).

Our findings raise important questions about the effectiveness of restoration efforts to date in peatlands, where many “restored” systems remained net CO_2_ sources, with 39% of individual restored sites functioning as net sources rather than sinks, similar to Kreyling et al. (2021). This suggests either insufficient recovery time, inappropriate restoration methods, legacy effects of degradation, or a combination of these factors (Table S1). The restoration of degraded peatlands employs a range of methods, from vegetation management to hydrological engineering, in order to re-establish vegetation cover and raise the water table, respectively (Andersen et al. 2016; Dohong et al. 2018; Waddington and Price 2013). However, carbon balance recovery following restoration depends on the complex interplay of numerous interacting factors, including land use, vegetation, above- and belowground biomass (Griffiths et al. 2018; Jordan et al. 2016; Webster et al. 2018), water table depth, peat and water physical and chemical properties (Couwenberg et al. 2009; Hirano et al. 2008; Kim and Verma 1992; Melling et al. 2017), surface pool area (Pelletier et al. 2015), air and soil temperatures, and available photosynthetic radiation (Blodau 2002; Bubier et al. 2003). Some studies in our analysis measured CO_2_ fluxes within only one to two years post-restoration interventions (Holl et al. 2019), a timeframe potentially insufficient for the vegetation re-establishment, peat structural recovery, and biogeochemical stabilization necessary to restore net carbon sink function (Petrone et al. 2003; Waddington and Price 2013). Furthermore, severely degraded peatlands with substantial peat loss (Jurasinski et al. 2020), such as the peat extraction studies in our analysis (Beyer and Höper 2015; D′Acunha et al. 2019; Minke et al. 2016), may require many decades to recover CO_2_ uptake capacity, while some may never return to net sink status (Bortoluzzi et al. 2006; Hambley et al. 2019). In addition, not all restoration techniques effectively reduce GHG emissions (Dixon et al. 2014), and some approaches (such as a rewetted fen nature reserve (Poyda et al. 2016) and a peatland within a national park (Hirano et al. 2008) that remained net CO_2_ sources with +280 and +174 gCO_2_-C m^-2^yr^-1^, respectively) may prioritize other objectives (biodiversity, hydrology) over immediate carbon benefits.

### Water Table Controls on NEE

Our analysis revealed a significant linear relationship between water table depth and annual NEE across peatlands globally, with CO_2_ emissions increasing by approximately 7.2 gCO_2_-C m^−2^ yr^−1^ for each centimeter of water table drawdown. The observed near-surface (−7.8 cm) transition aligns with critical water-table zones and hydrological tipping points reported for other peatlands [−14 to −17 cm; (Albert-Saiz et al. 2025b)], [−15 cm for fens and −20 cm for bogs; Albert-Saiz et al. (2025a)] and [~24 cm; Jassey et al. (2018)]. Typical drainage-affected degraded peatlands have lower water table levels, as most alternative peatland uses, including agriculture, forestry, and peat extraction, require water to be drained from the peatland (Gronlund et al. 2006; Jackson et al. 2009; Schrier-Uijl et al. 2010). Lowered water table levels expose upper peat layers to oxygen, initiating a rapid aerobic decomposition process (Jayasekara et al. 2024), which can release increased amounts of CO_2_ into the atmosphere (Carlson et al. 2012; Hatano 2019; Jayasekara et al. 2025b). When these enhanced respiratory losses exceed photosynthetic CO_2_ uptake by vegetation, the peatland transitions from net CO_2_ sink to source (Oertel et al. 2016; Schrier-Uijl et al. 2014). The water table-NEE relationship may help explain the patterns we observed in restored peatlands (Table S1), where 39% of restored sites remained net CO_2_ sources. Continuous CO_2_ emissions may suggest incomplete hydrological recovery, where restoration interventions failed to re-establish or maintain water tables sufficiently near the surface (e.g., Strack and Zuback (2012), Holl et al. (2019), Poyda et al. (2016) and Hambley et al. (2019). This emphasizes that successful peatland restoration requires active, sustained hydrological management, not merely revegetation or cessation of drainage, with water table targets based on reference conditions from intact systems that are net CO_2_ sinks (Strack and Waddington 2007; Waddington et al. 2001). Climate-driven water table decline, edge effects from adjacent drainage infrastructure, or subtle anthropogenic disturbances can shift even apparently undisturbed peatlands from carbon sinks to sources (Alfadhel et al. 2020; Euskirchen et al. 2012; Voigt et al. 2017), highlighting the sensitivity of these ecosystems to hydrological perturbations and the importance of landscape-scale water management.

### Limitations and Recommendations for Standardization of Data Collection and Processing

Our systematic review revealed substantial methodological differences in data collection and processing methods across the 174 studies examined. We identified three primary NEE measurement approaches and 22 different software programs used for data processing. The dominant methods, eddy-covariance (EC) and chamber-based measurements, differ fundamentally in their spatiotemporal scales (Fang and Moncrieff 1996; Poyda et al. 2017). EC systems provide continuous and direct ecosystem-scale measurements (typically 100-1000 m^2^ footprint) (Baldocchi 2003; Goulden et al. 1996; Loescher et al. 2006), while chamber methods measure small patches (0.01-1 m^2^) either continuously or intermittently (Fang and Moncrieff 1996; Law et al. 2001; Poyda et al. 2017), requiring spatial upscaling and temporal interpolation to estimate annual budgets (Acosta et al. 2019; Poyda et al. 2017). Most studies use one method for flux measurements, but the few that use more than one method identify a range of factors affecting the comparability of results. While some studies report similar flux estimates between methods when chambers adequately capture microtopographic variability (Flanagan and Johnson 2005; Schrier-Uijl et al. 2010; Wang et al. 2013), others document weak correlations between methods, due to noisy EC measurements at night and low turbulence wind conditions, particularly in heterogeneous peatland landscapes (Goulden et al. 1996; Lavigne et al. 1997; Podgrajsek et al. 2014; Wohlfahrt et al. 2005). Supporting findings of the former set of studies, one study in our analysis reported comparable CO_2_ flux estimates between methods when chambers were sufficiently replicated and strategically positioned to capture microtopographic variability (Laine et al. 2006). Although such studies indicate EC and chamber methods can yield fluxes of the same magnitude, the two methods may be best viewed as complementary, rather than fully comparable (Acosta et al. 2019; Podgrajsek et al. 2014).

Data processing approaches ranged from specialized EC software (EddyPro, TK3) to general statistical environments (R, Python, MATLAB) and basic spreadsheet calculations. This diversity in methodological approaches introduces inconsistencies across gap-filling algorithms, quality control protocols, uncertainty quantification, and particularly chamber upscaling, which requires spatial sampling strategies and temporal interpolation methods to capture peatland microtopography (Alm et al. 2007; Pumpanen et al. 2004; Shi et al. 2022). These methodological inconsistencies may have contributed to the high variability we observed in restored peatland CO_2_ fluxes (−397 to +1170 gCO_2_-C m^−2^ yr^−1^) and complicate the synthesis of restoration outcomes. Furthermore, the predominance of short-term studies (43 sites with only 1 year of data) limits the ability to understand interannual variability and long-term trends, underscoring the need for long-term CO_2_ flux monitoring in peatlands.

## Conclusions

This systematic review and meta-analysis establish a comprehensive quantitative synthesis of global peatland NEE across climatic zones, land uses, and peatland conditions, revealing several major findings with implications for carbon science and peatland management. Traditional peatland condition classifications based on land-use history or visual assessment inadequately capture functional carbon status. The frequent misclassification of intact sites as CO_2_ sinks and degraded sites as CO_2_ sources demonstrates that CO_2_ sink/source status exists along a continuum and cannot be reliably inferred from categorical condition labels alone. Water table depth emerged as the most significant driver of NEE across peatland types and climatic zones, confirming hydrological restoration as the primary lever for recovering carbon sink function in degraded systems. However, because raising the water table also promotes anaerobic conditions, restoration can increase methane emissions, and its net climate benefit should therefore be evaluated in terms of full greenhouse-gas balance rather than CO_2_ alone.

A severe geographic bias exists in peatland NEE research, with studies concentrated in Northern Hemisphere boreal and temperate regions. Tropical, African, and Oceanian peatlands, which are among the world’s most carbon-dense ecosystems, remain critically understudied, representing a major gap in global carbon budget estimates. Substantial methodological heterogeneity across studies, particularly in chamber-based measurements and data processing workflows, combined with predominantly short study durations, fundamentally limits cross-study comparability and the utility of NEE as a restoration monitoring tool. Collectively, these findings provide robust, evidence-based emission estimates to support national greenhouse gas inventories and UNFCCC reporting, while strengthening the scientific case for peatland conservation and restoration as nature-based climate solutions.

## Supplementary information

Online_Resources_1

Online_Resources_2_Database

## Data Availability

The database created and used for this study is included as an electronic supplementary material (Online_Resources_2_Database) with the submission.
