# Supplementary material for: Global Patterns of Net Ecosystem Exchange in peatlands: A Systematic Review and Meta-analysis of Drivers Across Land Use and Environmental Gradients
Source: Environ Manage. 2026 Sep 12;76(9):310. doi: 10.1007/s00267-026-02621-y (PMC13570830; doi:10.1007/s00267-026-02621-y)
Supplement: Supplementary file 1 [file 267_2026_2621_MOESM1_ESM.pdf]

Online Resources for the manuscript

Global patterns of Net Ecosystem Exchange in peatlands: A systematic review and meta-analysis of drivers across land use and environmental gradients

Charuni Jayasekara*[a,e,f], Catherine Leigh[b], Jeff Shimeta[c], Ewen Silvester[d], Samantha Grover[a]

[a]Department of Applied Chemistry and Environmental Science, School of Science, RMIT University, Melbourne, Victoria, Australia

[b]Queensland Herbarium and Biodiversity Science, Department of the Environment, Tourism, Science and Innovation, Brisbane, Queensland, Australia

[c]Department of Biology, School of Science, RMIT University, Bundoora, Victoria, Australia

[d]Research Centre for Applied Alpine Ecology, Department of Ecological, Plant and Animal Sciences, La Trobe University, Wodonga, Victoria, Australia

[e]School of Agriculture, Food and Ecosystem Sciences (SAFES), Faculty of Science, The University of Melbourne, Parkville, Victoria, Australia

[f]School of Biology and Environmental Science (BES), Queensland University of Technology, Brisbane, Queensland, Australia

*Corresponding author present contact: Charuni Jayasekara, Email: charuni.jayasekara@qut.edu.au, School of Biology and Environmental Science (BES), Queensland University of Technology, Brisbane, Queensland, Australia

Testing for a threshold in water table depth (WTD) and Net Ecosystem Exchange (NEE) relationship

Methods

To assess whether the relationship between annual net ecosystem exchange (NEE) and water-table depth contained a threshold rather than a continuous response, we conducted a segmented (breakpoint) regression fitted by iterative maximum likelihood (Muggeo 2003), using the *segmented* package (Muggeo 2008) in R version 4.4.1 (R Core Team 2020). We tested whether a breakpoint was statistically justified using Davies' test and a score-based test for a change in slope (Muggeo 2016), and compared the linear and segmented models using the Akaike and Bayesian information criteria (AIC and BIC). The stability of the estimated breakpoint was evaluated by (i) refitting the segmented model after excluding NEE outliers ($|z| > 3$) and (ii) a non-parametric bootstrap (3,000 resamples) of the breakpoint location. Analyses used the 98 sites for which water-table depth was reported. The water-table depth at which sites transitioned from net CO_2 sink to net source (i.e. the water-table position at NEE = 0) was estimated from the linear model as the ratio of the intercept to the slope, with a 95% confidence interval obtained by the delta method (car package; Fox and Weisberg (2018)).

Results

The linear model indicated a significant negative relationship between NEE and water-table depth (NEE = −56.0 − 7.18 × WTD; $r^2 = 0.19$, $F_{1,96} = 22.5$, $p < 0.001$), with sites transitioning from net CO_2 sink to net source close to the peat surface (water-table depth at NEE = 0: −7.8 cm; 95% CI −25.5 to +9.9 cm) (Figure S1). The segmented regression provided no evidence of a threshold (Figure S1.a). Neither the Davies test ($p = 0.54$) nor the score test ($p = 0.91$) indicated a significant change in slope, and both information criteria favoured the linear model over the segmented model (AIC: 1502.8 vs 1504.4; BIC: 1510.6 vs 1517.3). Although the segmented model returned a nominal breakpoint at −60.7 cm (Figure S1.b), its standard error was large (±24.9 cm), the slope below the breakpoint did not differ from zero (−0.59 ± 5.90 g CO_2-C m^{-2} yr^{-1} per cm; $p = 0.92$), and the estimate was not robust: excluding two NEE outliers shifted the breakpoint to +8.1 cm (±48.3 cm), and the bootstrap distribution of the breakpoint spanned essentially the entire drawdown range (95% CI −72 to 0 cm). NEE therefore responded continuously to water-table depth across the observed range, with no statistically defined threshold.

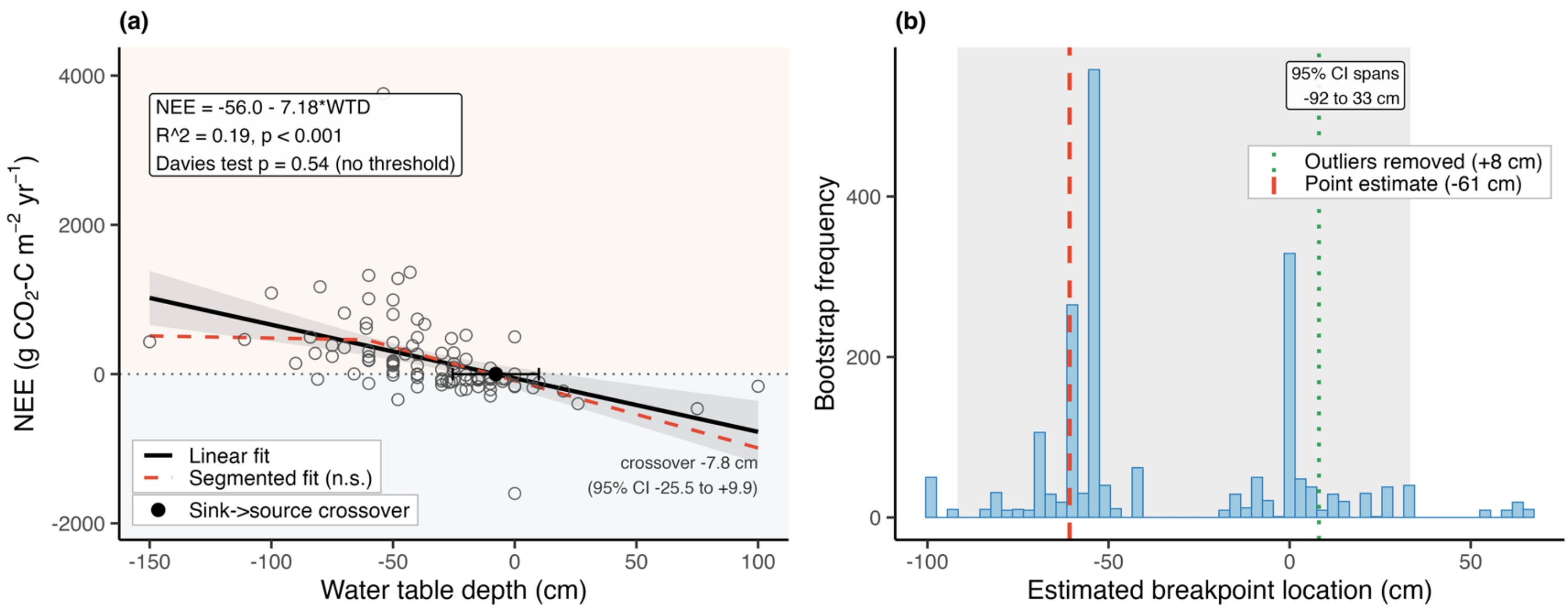

Fig. S1 Test for a threshold in the water-table–NEE relationship (n = 98). (a) Annual NEE against water-table depth with the linear fit (solid line, 95% confidence band) and the segmented (breakpoint) fit (dashed); the two are near-identical and the segmented model does not improve fit (Davies' test p = 0.54; score test p = 0.91; AIC and BIC both favouring the linear model). The point marks the sink-to-source crossover (water-table depth at NEE = 0: −7.8 cm, 95% CI −25.5 to +9.9 cm). Shading distinguishes net sink (below 0) from net source (above 0). (b) Bootstrap distribution (3,000 resamples) of the estimated breakpoint location, which spans the full drawdown range (95% CI −72 to 0 cm) and shifts to +8 cm when two outliers are excluded, indicating no well-defined threshold.

Table S1 Restoration history and annual net ecosystem exchange (NEE) of the 18 restored peatland sites in the dataset. Water table depth is negative below the peat surface. Negative NEE = net CO_2 sink, positive NEE = net CO_2 source.

Source study	Country	Prior degradation / land-use history	***Restoration intervention	Restoration timing (as recorded in source)	Years since restoration at measurement (approx.)	Water table depth (cm)	Annual NEE (g CO_2-C m^{-2} yr^{-1})	Net status
Beyer and Höper (2015)	Germany	Peat mining and agriculture	Rewetting	Rewetted after agricultural use in 2004	*7	-5	-98.7	Sink
Strack and Zuback (2012)	Canada	Cutover (peat harvesting)	Restoration of cutover peatland	Restored for 10 years	10	-27	111.1	Source
Hambley et al. (2019)	Scotland	Afforestation on blanket bog	Forest-to-bog restoration (rewetting)	10 years after restoration	10	-10	80.0	Source
Knox et al. (2015)	USA	Agriculture (drained pasture, Delta)	Rewetting / wetland re-establishment	Restored in 1999	*13	26	-397.0	Sink
Hambley et al. (2019)	Scotland	Afforestation on blanket bog	Forest-to-bog restoration (rewetting)	16 years after restoration	16	-10	-71.0	Sink
Bortoluzzi et al. (2006)	France	Peat harvesting	Regeneration of cutover bog	Restoration started 20 years ago	20	-10	-126.8	Sink
**Poyda et al. (2016)	Germany	Agriculture (fen, forage)	Rewetting (nature-reserve / extensive fen)	Rewetting started ~two decades ago	*20	-30	280.0	Source
**Poyda et al. (2016)	Germany	Agriculture (fen, forage)	Rewetting	Rewetting started ~two decades ago	*20	-50	800.0	Source
**Poyda et al. (2016)	Germany	Agriculture (fen, forage)	Rewetting	Rewetting started ~two decades ago	*20	-60	1010.0	Source
**Poyda et al. (2016)	Germany	Agriculture (fen, forage)	Rewetting	Rewetting started ~two decades ago	*20	-80	1170.0	Source
Beyer and Höper (2015)	Germany	Peat mining	Rewetting of former bog peat-extraction site	Rewetted after peat mining in 1983	*27	-15	-71.4	Sink

Chamberlain et al. (2018)	USA	Agriculture (drained pasture, Delta)	Rewetting / wetland re-establishment	Restored in 2010	*7	100	-3.4	Sink (near-neutral)
Holl et al. (2020)	Germany	Drained / degraded fen	Rewetting	Started rewetting (date not reported)	not reported	-20	241.0	Source
Minke et al. (2016)	Belarus	Peat mining (milling)	Rewetting / inundation of cutover fen	not reported	not reported	0	-169.0	Sink
Minke et al. (2016)	Belarus	Peat extraction + grassland	Rewetting / inundation of cutover fen	not reported	not reported	20	-227.7	Sink
D′Acunha et al. (2019)	Canada	Farming + peat mining (delta bog)	Rewetting of peat bog	not reported	not reported	-15	-57.8	Sink
Bortoluzzi et al. (2006)	France	Peat harvesting	Regeneration of cutover bog (contrasting stage)	not reported	not reported	-25	26.5	Source
Nugent et al. (2018)	Canada	Moss (Sphagnum) harvesting	Restoration after moss harvesting	not reported	not reported	-28	-90.0	Sink

Notes:
*"Years since restoration" is taken directly from the source article where stated are approximate, derived by differencing the restoration year against the measurement year.

**The four Poyda et al. (2016) sites were all rewetted ~20 years before measurement; NEE increases with water-table drawdown across them, illustrating that at comparable recovery time, hydrological condition governs sink/source status.

***Interventions are dominated by rewetting; the sample does not support a formal statistical analysis and is presented descriptively.

References

Beyer C, Höper H (2015) Greenhouse gas exchange of rewetted bog peat extraction sites and a *Sphagnum* cultivation site in northwest Germany. *Biogeosciences*, *12*(7), 2101-2117. https://doi.org/10.5194/bg-12-2101-2015

Bortoluzzi E, Epron D, Siegenthaler A, et al. (2006) Carbon balance of a European mountain bog at contrasting stages of regeneration. *New Phytol*, *172*(4), 708-718. https://doi.org/10.1111/j.1469-8137.2006.01859.x

Chamberlain SD, Anthony TL, Silver WL, et al. (2018) Soil properties and sediment accretion modulate methane fluxes from restored wetlands. *Glob Chang Biol*, *24*(9), 4107-4121. https://doi.org/10.1111/gcb.14124

D'Acunha B, Morillas L, Black TA, et al. (2019) Net Ecosystem Carbon Balance of a Peat Bog Undergoing Restoration: Integrating CO_2 and CH_4 Fluxes From Eddy Covariance and Aquatic Evasion With DOC Drainage Fluxes. *Journal of Geophysical Research: Biogeosciences*, *124*(4), 884-901. https://doi.org/10.1029/2019jg005123

Fox J, Weisberg S (2018) *An R companion to applied regression* (3 ed.). Sage publications.

Hambley G, Andersen R, Levy P, et al. (2019) Net ecosystem exchange from two formerly afforested peatlands undergoing restoration in the Flow Country of northern Scotland. *Mires and Peat*, *23*(5), 1-14. https://doi.org/10.19189/MaP.2018.DW.346

Holl D, Pfeiffer E-M, Kutzbach L (2020) Comparison of eddy covariance CO2 and CH4 fluxes from mined and recently rewetted sections in a northwestern German cutover bog. *Biogeosciences*, *17*(10), 2853-2874. https://doi.org/10.5194/bg-17-2853-2020

Knox SH, Sturtevant C, Matthes JH, et al. (2015) Agricultural peatland restoration: effects of land-use change on greenhouse gas (CO_2 and CH_4) fluxes in the Sacramento-San Joaquin Delta. *Global Change Biology*, *21*(2), 750-765. https://doi.org/10.1111/gcb.12745

Minke M, Augustin J, Burlo A, et al. (2016) Water level, vegetation composition, and plant productivity explain greenhouse gas fluxes in temperate cutover fens after inundation. *Biogeosciences*, *13*(13), 3945-3970. https://doi.org/10.5194/bg-13-3945-2016

Muggeo VM (2003) Estimating regression models with unknown break-points. Statistics in medicine. *Statistics in medicine*, *22*(1), 3055-3071. https://doi.org/10.1002/sim.1545

Muggeo VM (2008) Segmented: an R package to fit regression models with broken-line relationships. *R news*, *8*(1), 20-25.

Muggeo VM (2016) Testing with a nuisance parameter present only under the alternative: a score-based approach with application to segmented modelling. Journal of Statistical Computation and Simulation, 86(15), pp.3059-3067. *Journal of Statistical Computation and Simulation*, *86*(15), 3059-3067. https://doi.org/10.1080/00949655.2016.1149855

Nugent KA, Strachan IB, Strack M, et al. (2018) Multi-year net ecosystem carbon balance of a restored peatland reveals a return to carbon sink. *Global Change Biology*, *24*(12), 5751-5768. https://doi.org/10.1111/gcb.14449

Poyda A, Reinsch T, Kluß C, et al. (2016) Greenhouse gas emissions from fen soils used for forage production in northern Germany. *Biogeosciences*, *13*(18), 5221-5244. https://doi.org/10.5194/bg-13-5221-2016

R Core Team (2020) *R: A language and environment for statistical computing.* In https://www.R-project.org/

Strack M, Zuback YCA (2012) Annual carbon balance of a peatland 10 yr following restoration. *Biogeosciences Discussions*, *9*(5), 17203–17233. https://doi.org/10.5194/bgd-9-17203-2012
